# Double Burden of Malnutrition in Rural Madagascar: A Study on Infant Health in Ampefy

**DOI:** 10.3390/nu17111756

**Published:** 2025-05-22

**Authors:** Rosita Rotella, Jose M. Soriano, Agustin Llopis-Gonzalez, María Morales-Suarez-Varela

**Affiliations:** 1 Research Group in Social and Nutritional Epidemiology, Pharmacoepidemiology and Public Health, Department of Preventive Medicine and Public Health, Food Sciences, Toxicology and Forensic Medicine, Faculty of Pharmacy, Universitat de València, Av. Vicent Andrés Estelles s/n, 46100 Burjassot, Valencia, Spain; rotella@alumni.uv.es (R.R.); agustin.llopis@uv.es (A.L.-G.); 2 Centro Medico-Chirurgico Saint Paul (Change ONG), Andasibe, Ampefy 118, District of Soavinandriana, Itasy Province, Madagascar; 3 Observatory of Nutrition and Food Safety for Developing Countries, Food & Health Lab, Institute of Materials Science, University of Valencia, Carrer Catedrático Agustín Escardino, 9, 46980 Paterna, Spain; jose.soriano@uv.es; 4 Joint Research Unit on Endocrinology, Nutrition and Clinical Dietetics, University of Valencia-Health Research Institute La Fe, Avda. Fernando Abril Martorell, 106, 46026 Valencia, Spain; 5 Biomedical Research Center in Epidemiology and Public Health Network (CIBERESP), Carlos III Health Institute, Av. Monforte de Lemos 3-5. Pabellón 11, Planta 0, 28029 Madrid, Spain

**Keywords:** malnutrition, infant, Madagascar

## Abstract

**Background/Objectives**: This study investigates the prevalence and impact of the double burden of malnutrition (DBM), malnutrition and overnutrition, in infants aged 0–24 months in the rural municipality of Ampefy, Itasy region, Madagascar. The Global Nutrition Report 2022 highlights the widespread issue of malnutrition, affecting 2 billion adults and 38 million children under five. Madagascar, characterized by severe poverty and high malnutrition rates, serves as a critical case study. **Methods**: A structured questionnaire was administered to 437 mother–child pairs from November 2022 to March 2023, collecting data on maternal education, dietary habits, and socio-economic status. Anthropometric measurements were taken using either a stadiometer or UNICEF length charts to assess height/length and an electronic scale to determine weight. **Results**: Findings reveal a high prevalence of malnutrition, with 29.7% of children affected by either wasting, stunting, and/or underweight, and 13.3% classified as overweight or obese. This study identifies significant age-related differences, with younger children more likely to be overweight and older children more likely to suffer from stunting or wasting. Maternal nutritional status, breastfeeding practices, and socio-economic conditions are strongly associated with child malnutrition outcomes. Lower dietary diversity among mothers and insufficient antenatal care are significant risk factors. Households with lower incomes and limited access to water and transport face higher malnutrition risks. **Conclusions**: This study underscores the critical need for targeted nutritional interventions and education to address DBM in Madagascar, highlighting the importance of maternal health and dietary diversity in early childhood development. Further longitudinal research is recommended to establish causality and develop comprehensive public health strategies.

## 1. Introduction

The latest Global Nutrition Report (2022) indicated that approximately 2 billion adults are either overweight or affected by obesity [1]. Additionally, it is estimated that 38 million children under the age of five are overweight, 149 million are stunted, and 45 million are wasted. Malnutrition is a global health problem that affects almost all individuals, regardless of age, gender, race, social status, and geographical boundaries [2]. It can be defined as an imbalance in energy and nutrient intake that can alter the body’s measurements, composition, and functions [3], and it can be divided into three principal categories of malnutrition: underweight, overweight, and micronutrient-related malnutrition (MRM). The three most prevalent forms of malnutrition are stunting, wasting, and underweight. The growing attention devoted to this public health issue has prompted a shift in focus from a predominant emphasis on single forms of malnutrition to a comprehensive approach encompassing all manifestations of malnutrition. This shift has led to the introduction of the concept of the double burden of malnutrition (DBM), which refers to the coexistence of two distinct but interrelated challenges: malnutrition, characterized by micronutrient deficiencies, underweight, stunting, and wasting in childhood, and the rise of overweight, obesity, and diet-related non-communicable diseases associated with overnutrition [4].

DBM represents a significant global health challenge, with evidence indicating a high prevalence in low- and middle-income countries (LMICs). A significant proportion of LMICs are affected by high rates of underweight and overweight. In over one-third of these countries, more than 30% of children are stunted, more than 15% are wasted, and more than 20% are underweight or thin. Additionally, more than 20% of adults are overweight [5]. Within this context, Madagascar is one of the countries where the prevalence of malnutrition still represents an emergency, particularly in rural areas [6]. A majority of these malnourished individuals are extremely impoverished and encounter significant challenges due to political instability, constrained economic growth, and inadequate access to fundamental services. The exacerbation of the malnutrition issue is further compounded by economic disparities and constrained access to healthcare. As reported by the World Food Programme [7], 92% of the Malagasy population subsists on less than USD 1.90 per day, with approximately half of all children under the age of five affected by stunting. At the national level, Madagascar reports a high prevalence of stunting, with the Itasy region exhibiting the highest rates at 62.3%. The highest prevalence of stunting is observed in children aged 18–23 months [8].

The occurrence of malnutrition during pregnancy, which exerts an influence on fetal growth and the first two years of life, represents a significant determinant of stunting, obesity and non-communicable diseases in adulthood [9]. The majority of growth arrest occurs within the first 1000 days following conception. This period represents a critical window of opportunity for optimal physical and cognitive development. Furthermore, optimal growth during the first 1000 days of life is crucial for the prevention of overweight. While weight attained at any age in the first few years of life is positively associated with adult body mass index in cohorts from less developed countries, rapid weight gains in the first 1000 days are strongly associated with adult lean mass, while weight gains later in childhood lead mainly to adult fat mass. In particular, evidence indicates that infants who experienced stunted growth during the first few years of life and subsequently gained weight rapidly later in childhood may be at an elevated risk of developing obesity and non-communicable diseases in adulthood [10,11,12].

The objective of this study is to assess the prevalence of the double burden of malnutrition at the community level among the infant population (0–24 months) in a rural municipality in the highlands of Madagascar. This manuscript presents findings on the factors and indicators associated with nutritional status, including women’s knowledge and practices during pregnancy, the socio-economic status of households in the intervention area, dietary quality, and anthropometric measurements of mother–child pairs in the study population.

## 2. Materials and Methods

### 2.1. Study Design and Area

This exploratory, observational, and analytical mother–child study was conducted from 1 November 2022 to 31 March 2023, in Ampefy, Itasy region, Madagascar. Data collection involved a structured questionnaire survey of women residing in the municipality of Ampefy who had live children aged 0–24 months. The interview included collecting the mother’s medical history and recording anthropometric parameters for both the mother and child. Ampefy is a rural municipality with a population of 25,078, divided into 13 smaller administrative units known as fokontanies. These units represent the smallest administrative divisions in Madagascar and comprise groups of small settlements. The population of this region is diverse, with various Malagasy cultural and ethnic groups, though the Merina ethnic group predominates. The region experiences a subtropical climate heavily influenced by monsoons. The rainy season, typically from November to March, often brings destructive floods, hindering agricultural production and food security. Conversely, the dry season, from May to September, is marked by a lack of rainfall, which similarly affects agriculture, exacerbating existing food insecurity and malnutrition issues. Madagascar ranks among the nations most impacted by the climate crisis [13,14].

### 2.2. Study Population and Data Collection

The study population was identified by Change Onlus staff using the patient registry of the St. Paul’s Medical and Surgical Centre, the NGO’s base. Women were included if they met the following criteria: (i) reproductive age (15–49 years), (ii) had at least one live child aged 0–24 months, and (iii) were breastfeeding at least one child aged 0–24 months. Exclusion criteria were (i) lack of informed consent, (ii) unreliable responses, and (iii) incomplete questionnaire responses. A systematic random sampling method was used to invite eligible women from health-center registers. Women who visited the health center with their children or utilized its services on days when project personnel were present were selected.

Demographic data indicated that approximately 5000 women of reproductive age (15–49 years) lived in Ampefy, and 500 were selected to be invited to participate. Of the 500, 437 were able to be contacted and invited to participate. All invited women accepted the invitation and gave informed consent, resulting in the inclusion of 437 mothers with breastfed children aged 0–24 months in the study. Data collection commenced in November 2022 and concluded in March 2023. A structured questionnaire was employed, and the research team underwent a seven-day training session on data collection techniques, including discussions and tests of study procedures and tools with a group of non-participant mothers. Data were collected in the 13 fokontanies in Ampefy and at the NGO’s health center headquarters. The questionnaire, administered through face-to-face interviews, lasted approximately 20 min per participant. Anthropometric measurements of the mother and child were recorded, and the mother’s medical history was reviewed if available. Participants were informed about the study’s objectives and the confidentiality of the data. Informed-consent documents were orally translated by local health-center workers. Each participant received an identification number to anonymize the data, ensuring confidentiality. The questionnaires were administered using tablets.

### 2.3. Ethical Considerations

The study was approved by the Ethics Committee of the Universitat de Valencia (Spain) (protocol code Register code: 2089516, approved on 7 July 2022) and the Ethics Committee of the St. Paul Medical–Surgical Center and the Institut d’Enseignement Superieur de Soavinandriana, Universitè d’Antananarivo (Madagascar) (protocol code Register code: 20220197, approved on 27 October 2022). The study adhered to the guidelines of the World Medical Association’s Declaration of Helsinki and other relevant ethical guidelines for medical research involving human subjects and children.

### 2.4. Tools of Measurement

The semi-structured, non-pre-tested questionnaire aimed to collect data on maternal education levels; attitudes towards antenatal care; dietary habits during pregnancy and lactation; and the relationship between micronutrient intake, dietary diversity, and socio-demographic variables. Initially formulated in English, the questionnaire was translated into Malagasy by a local translator proficient in English. A back-translation was conducted to ensure accuracy. Ten mothers from the health center reviewed the questionnaire for feedback on its accuracy.

Based on pilot feedback, the number of questions was reduced due to the observed decrease in women’s attention span after 25 min. Questions regarding personal hygiene and the father’s role in feeding decisions were eliminated. Mothers who participated in the pilot study were excluded from the main study to prevent bias.

The questionnaire comprised four principal sections: (i) general characteristics of the mothers, including age, education level, occupation, and number of pregnancies; (ii) practices during pregnancy and breastfeeding, such as antenatal care, place of birth, breastfeeding, and integration; (iii) living conditions, including health status, access to drinking water, proximity to health centers, and availability of transport; and (iv) Minimum Dietary Diversity for Women of Reproductive Age (MDD-W) assessment, based on dietary intake in the 24 h preceding the interview.

The MDD-W assessment used a list-based technique to record dietary diversity within households. Interviewers presented a culturally adapted list of food items to participants. Data collectors were trained to categorize meals containing mixed food groups and to record only food groups where more than 15 g was consumed. The Dietary Diversity Score (DDS) was calculated using 10 food groups, as suggested by the FAO for women of reproductive age.

The anthropometric assessments were conducted by experienced interviewers with extensive training in anthropometric measurements and seven years of experience in the Child Malnutrition Prevention and Treatment Project of Change Onlus.

Maternal measurements were taken using a SECA stadiometer and an electronic scale (SECA Clara 803, Hamburg, Germany), with height and weight recorded to the nearest 0.1 cm and kg, respectively. Two height measurements were averaged to minimize error. BMI was calculated and categorized according to WHO. Two measurements were taken for each mother’s height, and the average was calculated to minimize measurement error. The mother’s BMI was calculated by dividing her weight by the square of her height (kg/m^2^), and her nutritional status was classified according to WHO cut-off values: underweight (BMI < 18.5 kg/m^2^), normal weight (18.5 ≤ BMI < 24.9 kg/m^2^), and overweight (BMI ≥ 25.0 kg/m^2^).

UNICEF length charts were used to measure the child’s length in the prone position, with an accuracy of 0.1 cm. The child’s weight was measured using the same electronic scale used for maternal weight and was determined by calculating the difference between the weight of the mother and the weight measured while the mother held her child. The child’s positioning during the measurements was supervised by the interviewer, with assistance from the child’s mother and a nutritionist employed by the NGO to ensure reliability.

Nutritional status was determined using WHO cut-off points for weight for height (WFH), height for age (HFA), weight for age (WFA), and body mass index for age (BMIFA). Wasting was identified by a WFH score below −2, stunting by an HFA score below −2, and underweight by a WFA score below −2. Overweight and obesity were determined by a BMIFA > +1 z-score. All raw anthropometric data were entered into WHO Anthroplus software to calculate HFA, WFA, and BMIFA, while an online tool was used to calculate WFH.

### 2.5. Data Analysis

The characteristics of mothers and children were analyzed using descriptive statistics, presented as means and standard deviations, medians, and interquartile ranges, or as frequencies and percentages. Normality of the distribution was assessed using the Kolmogorov–Smirnov test. Comparisons of variables between stunted or wasted or underweight, and overweight or obese children were made using the Chi-square test or McNemar test for qualitative variables and ANOVA for quantitative variables. Statistical significance was set at α < 0.05. All analyses were performed using IBM SPSS Statistics version 26.0 (IBM SPSS Statistics, Chicago, IL, USA).

## 3. Results

The general characteristics and profile of the children in the study are presented in Table 1. Of the total sample of 437 children, 130 (57.0%) were affected by wasting (WFA z-score < −2), stunting (HFA z-score < −2), or underweight (WFL z-score < −2); 58 (13.3%) were classified as overweight or obese (BMIFA z-score > +1), and 249 had z-scores within the normal range. Age showed statistically significant differences among the three groups. Children who were wasted, stunted, or underweight had a mean age of 12.92 ± 5.58 months, whereas children who were overweight or obese had a mean age of 4.53 ± 4.90 months. Children with normal z-scores had a mean age of 10.68 ± 6.37 months. Notably, overweight/obese infants were significantly younger, with a mean age of 4.53 months, compared to 10.53 months for healthy weight and 10.68 months for stunted/wasted/underweight infants. This early onset of overweight/obesity underscores the critical need for early intervention. In terms of sex distribution, males are slightly more prevalent in the stunted/wasted/underweight group, though the differences are not statistically significant. Birth weight also plays a crucial role; low birth weight (<2.5 kg) is more common among stunted/wasted/underweight infants, with an odds ratio (OR) of 2.05, highlighting the association between low birth weight and poor growth outcomes. Anthropometric measurements reveal that overweight/obese infants have a lower mean weight and height but higher mid-upper arm circumference (MUAC) compared to their healthy weight and stunted/wasted/underweight peers. Specifically, the MUAC measurements show that 43.1% of stunted/wasted/underweight infants fall into the moderate acute malnutrition (MAM) category, compared to just 6.8% of healthy weight infants, indicating severe malnutrition issues.

The general characteristics of the mothers are presented in Table 2. Anthropometric parameters of the mothers also show statistically significant differences among the three groups. Women with a BMI below 18.5 kg/m^2^ were more prevalent in the group with stunted, wasted, or underweight children (31.5%), while women with a BMI above 24.9 kg/m^2^ were more common among those with overweight or obese children. The mean BMI for mothers of healthy weight children was 20.77 kg/m^2^, compared to 20.95 kg/m^2^ for mothers of stunted/wasted/underweight children, and 21.29 kg/m^2^ for mothers of overweight/obese children (*p* = 0.025). Educational level did not show statistically significant differences among the groups (*p* = 0.060). However, a primary-education level or lower was more common among mothers of stunted, wasted, or underweight children (53.1%) compared to those with healthy weight (40.6%) or overweight/obese children (48.3%). No significant differences were observed in caregiving practices among the groups. However, mothers of stunted, wasted, or underweight children had fewer contacts with healthcare personnel during pregnancy and took iron and folic acid supplements less frequently than other mothers. A higher proportion of this group gave birth at home. A pregnancy interval of less than 24 months was a significant risk factor; 9.2% of children with stunting, wasting, or underweight had mothers with a previous pregnancy within two years, compared to 5.6% in children with normal z-scores, and none in overweight/obese children, indicating a statistically significant difference (*p* = 0.0185). Similarly, optimal breastfeeding practices were less frequently adhered to by mothers of children with stunting, wasting, or underweight. Attention to diet during pregnancy showed statistically significant differences among the groups, with a lower frequency of proper nutritional care observed in the group of children suffering from stunting, wasting, or underweight.

The general characteristics of the mothers and their maternal care and feeding practices are detailed in Table 3. The data indicate significant differences among mothers with healthy-weight children, those with stunted/wasted/underweight children, and those with overweight/obese children. For antenatal care, 90.6% of mothers attended four or more visits, with no significant differences among the groups. Iron and folic acid supplementation during pregnancy was common, with 83.8% of mothers adhering to this practice. Vaginal delivery was the predominant mode of birth (92.0%), with operative vaginal births and cesarean sections being less common. Home births were notably higher among mothers of stunted/wasted/underweight children (59.2%) compared to those of healthy weight children (34.1%) and overweight/obese children (32.8%). This trend suggests potential barriers to accessing healthcare facilities. Early breastfeeding initiation within the first hour after birth was reported by 57.4% of mothers, with a slight decrease in the stunted/wasted/underweight group. Exclusive breastfeeding for the first six months was practiced by 44.2% of mothers, while breastfeeding continued up to one year in 96.1% of cases. Early weaning before six months was more prevalent among mothers of overweight/obese children (72.7%). Maternal diet improvements during pregnancy were reported by 53.8% of mothers, with a lower rate in the stunted/wasted/underweight group (44.6%). This highlights the importance of maternal nutrition on child health outcomes. Use of iodized salt was consistent across groups, with 75.5% adherence.

Table 4 presents the socio-economic characteristics of the households participating in the study, highlighting a generally uniform socio-economic status across the population. However, some significant differences were observed. Families with children affected by stunting, wasting, or underweight had a higher prevalence of households with an income below MGA 200,000. Both the stunting/wasting/underweight group and the overweight/obese group faced greater difficulty accessing the nearest water source, with an average walking time of more than five minutes in 53.8% and 53.4% of cases, respectively, a statistically significant difference (*p* = 0.034) compared to the healthy weight group. Family size was larger in households with overweight/obese children (mean 5.05) compared to other groups. Overcrowding, defined as less than 5 m^2^ of floor area per person, was more prevalent in the stunted/wasted/underweight group (53.8%). Middle-income levels were lower among stunted/wasted/underweight households, with 79.2% earning below MGA 200,000. Access to transportation was notably lower in the overweight/obese group (13.8%), with significant differences among the three groups (*p* = 0.048).

Table 5 presents the results related to the MDD-W. The group of children affected by stunting, wasting, or underweight showed lower dietary diversity scores among their mothers compared to the other groups. Specifically, 38.5% of mothers in this group consumed food from fewer than five different food groups, which is the standard threshold for dietary diversity. This indicates a less varied diet in this group. Overall, the MDD-W scores were lowest in the stunting, wasting, or underweight group. This group also had the highest prevalence of mothers not meeting the minimum dietary diversity. Although these mothers consumed plant-based proteins more frequently (38.5% pulses), their intake of animal-based proteins such as meat, poultry, fish (58.5%), and dairy products (26.9%) was significantly lower compared to other groups. In contrast, mothers of overweight/obese children had higher frequencies of meat, poultry, and fish consumption (79.3%) but still faced dietary diversity issues, with 31.0% not meeting the minimum dietary diversity threshold.

## 4. Discussion

The DBM is a complex global health issue, particularly in LMICs, including Madagascar. DBM refers to the coexistence of under and overweight within the same population, posing significant challenges for public health interventions. It is directly associated with the incidence of non-communicable diseases, creating a multifaceted problem that requires comprehensive strategies [15].

Our findings reveal a higher prevalence of undernutrition, including stunting, wasting, and underweight, compared to overweight and obesity. The prevalence of these conditions varies significantly with age (*p* < 0.001); overweight and obesity are more common in younger children (mean age of 4.53 ± 4.90 months), while stunting, wasting, and underweight are more prevalent in older children (mean age of 12.92 ± 5.58 months). This age-related difference underscores the importance of early nutritional interventions.

The protective effect of breastfeeding against malnutrition is well-documented. In the population under study, breastfeeding is widely practiced and continued up to the age of two, with very few exceptions—typically in cases of maternal illness. During the first months of life, infants benefit from exclusive breastfeeding, which, despite being relatively low in energy and micronutrients, is biologically tailored to their needs. Breastfeeding provides essential nutrients and protects infants against infections and diseases [16,17,18,19]. Even when mothers are undernourished, their bodies prioritize milk production, ensuring that the child receives adequate nutrients to maintain a normal weight status in early life [16]. Numerous studies have highlighted the benefits of breastfeeding, particularly in the first six months of life [16]. During this critical period, breast milk supplies all necessary nutrients for optimal growth and development, significantly reducing the risk of stunting, wasting, and underweight [20].

The success of breastfeeding is influenced by social, economic, and nutritional factors. Social limitations to women’s autonomy, employment, and maternal nutritional status can impact breastfeeding practices. Among mothers with severe underweight, low breast milk volume, and poor micronutrient status can adversely affect the growth and micronutrient status of their offspring. A poor maternal diet reduces the diversity of the maternal microbiome, which is passed on to the offspring, increasing the risk of severe underweight [4].

After six months, infants’ nutritional needs change, and breast milk alone becomes insufficient. This transition phase coincides with the introduction of complementary foods. While breastfeeding continues into the first year of life in 97.6% of cases, appropriate complementary feeding practices are crucial to reduce stunting incidence [21]. In this study setting, structural causes of malnutrition—such as widespread poverty, food insecurity, low dietary diversity, and limited knowledge of age-appropriate feeding practices—contribute to the inability of families to meet the growing energy and nutrient needs of their children. This results in a progressive deterioration of nutritional status, with underweight becoming more prevalent among older children.

Stunting, with its high prevalence among children in Madagascar, presents a dual challenge. It is associated with adverse developmental outcomes, including poor physical and cognitive development [22,23,24,25,26]. Furthermore, stunting increases the risk of developing obesity and non-communicable diseases in adulthood [27,28].

Malnutrition often spans generations, with critical periods of susceptibility during pregnancy and lactation. The maternal phenotype significantly influences early development [29]. Maternal anthropometry, particularly maternal height, is strongly associated with children’s malnutrition status, consistent with scientific evidence identifying maternal height as a determinant of stunting incidence [30]. Early growth fluctuations have long-term health implications. The thrifty phenotype hypothesis suggests that poor fetal nutrition reduces the growth of some organs (e.g., pancreas, liver, and kidney) to protect the brain. Such individuals are more likely to develop diseases related to obesity and energy-dense diets later in life, increasing the risk of non-communicable diseases [28,31,32].

Our study found no strong and consistent association between socio-economic status and all nutritional outcomes, aligning with other studies [33]. However, factors such as the mother’s occupation, average income, household size, and lack of sanitation were associated with an increased likelihood of stunting in several studies. Mothers of children classified as stunted, wasted, or underweight consumed animal protein less frequently than other groups. Reduced maternal nutrition during pregnancy and lactation further exacerbates this issue [34].

On the other hand, Madagascar’s diverse and abundant natural resources present opportunities to improve the well-being of the local population, especially in rural areas with limited food access. This study serves as a foundation for future educational interventions, addressing significant public health issues that affect vulnerable populations, including women, infants, and children. A woman’s health and nutritional status during fetal life, infancy, and the periconceptional period are closely linked to her pregnancy outcomes and the short- and long-term health of her offspring [35].

Early nutritional interventions are crucial in addressing both under- and overweight. Programs focusing on maternal nutrition, breastfeeding promotion, and timely introduction of complementary foods can significantly reduce the prevalence of stunting and wasting. Interventions such as micronutrient supplementation, provision of fortified foods, and education on dietary diversity are essential components of comprehensive strategies to combat malnutrition [21,36,37,38]. Maternal nutrition directly influences fetal and infant outcomes. Adequate maternal nutrition during pregnancy ensures proper fetal growth and development, reducing the risk of low birth weight, preterm birth, and subsequent stunting. Nutritional interventions targeting pregnant women, including iron and folic acid supplementation, balanced energy protein supplementation, and food fortification, have been shown to improve birth outcomes and reduce neonatal mortality [39,40]. Complementary feeding practices play a vital role in preventing malnutrition during the critical window of the first 1000 days of life. Introducing nutrient-rich complementary foods at six months, along with continued breastfeeding, supports optimal growth and development. Educational programs for caregivers on appropriate complementary feeding practices, hygiene, and food safety are essential to ensure the effectiveness of these interventions [41,42,43]. Addressing socio-economic determinants of malnutrition is critical for sustainable improvements in nutritional status. Interventions aimed at improving household food security, access to healthcare, sanitation, and education can create an enabling environment for better nutritional outcomes. Cash-transfer programs, agricultural interventions, and livelihood support can enhance food security and reduce poverty, contributing to improved maternal and child nutrition [44].

According to community-based approaches, integrated community-based programs that combine health, nutrition, and social interventions have shown promising results in reducing malnutrition. These programs often involve community health workers who provide nutrition education, monitor growth, and facilitate access to health services. Community engagement and participation are crucial for the success and sustainability of these programs [45,46]. Empowering women through education, income-generating activities, and leadership opportunities can significantly impact nutritional outcomes. Women’s empowerment enhances their ability to make informed decisions about their health and nutrition, leading to better outcomes for themselves and their children. Programs that promote gender equality and women’s rights are essential components of comprehensive nutrition strategies [47].

According to policy and programmatic implications, addressing DBM requires multisectoral approaches that integrate nutrition with health, agriculture, education, social protection, and economic development. National policies and programs should adopt a holistic approach, ensuring coordination and collaboration across sectors to address the underlying determinants of malnutrition. Strong political commitment and leadership are essential to drive these efforts [48]. Strengthening health systems to deliver quality nutrition services is critical for addressing malnutrition. This includes training healthcare providers, improving supply chains for essential nutrition commodities, and enhancing data systems for monitoring and evaluation. Health systems should be equipped to provide comprehensive maternal and child nutrition services, including antenatal care, growth monitoring, and treatment of severe acute malnutrition [49].

Focusing on the research and evidence gaps, longitudinal studies are needed to understand the long-term effects of early nutrition interventions on health and development outcomes. Research should focus on the impact of maternal and child nutrition programs on cognitive development, school performance, and economic productivity. Understanding the pathways through which nutrition influences long-term outcomes can inform the design of effective interventions [50]. Implementation research is essential to identify the best practices for scaling up nutrition interventions and integrating them into health systems. Studies should examine the feasibility, acceptability, and cost-effectiveness of different delivery models in diverse settings. Implementation research can provide insights into how to overcome barriers and optimize the delivery of nutrition services [51].

### Strengths and Limitations

This study has several notable strengths. This study employed a consistent sample size of 437 mother–child pairs, representing the first investigation, to our knowledge, performed to assess the impact of the double burden of malnutrition in Madagascar. The rigorous data collection process, which included the administration of a structured questionnaire and anthropometric measurements conducted by trained professionals, ensured the highest standards of data quality and minimized the potential for measurement error. By examining nutritional status from the moment of conception to the child’s second birthday, the study captures a critical period in child development and provides valuable insights into the field of early childhood nutrition. The study population encompassed a diverse range of cultural and ethnic groups in Madagascar, facilitating a comprehensive understanding of nutritional practices across different cultural contexts. Moreover, the study was conducted in accordance with the highest ethical standards and received approval from the ethics committees of the University of Valencia (Spain) and the Institut d’Enseignement Supérieur de Soavinandriana, Université d’Antananarivo (Madagascar). This ensures that the highest ethical standards were maintained throughout the study.

It should be noted, however, that this study is not without limitations. As an observational study conducted over a relatively short period, it is not possible to establish causality between nutritional status and outcomes. Longitudinal studies are required to understand the long-term effects of malnutrition in early childhood. It should be noted that some of the data, particularly those related to maternal diet and socio-economic status, were self-reported, thus introducing the possibility of reporting bias and imprecision. While the study provides valuable insights for the Itasy region, it is important to recognize that its results may not be generalizable to other regions of Madagascar or other countries with different nutritional status.

## 5. Conclusions

The study highlights the complex nature of the double burden of malnutrition (DBM) in Ampefy, Madagascar, revealing significant public health challenges. Underweight is prevalent among older children, while overweight and obesity are more common in younger infants, emphasizing the need for early nutritional interventions. Breastfeeding provides protective effects against malnutrition, but complementary feeding is crucial after six months. Socio-economic disparities, limited healthcare access, and environmental challenges contribute significantly to malnutrition. Maternal nutrition and dietary practices are critical in determining children’s nutritional outcomes, with mothers of undernourished children having lower dietary diversity and less frequent animal-based protein consumption. Barriers to healthcare, such as high home birth rates among undernourished children’s mothers, highlight the need for improved healthcare services. Educational interventions to improve maternal nutrition and feeding practices are essential. Addressing DBM requires a multisectoral approach integrating health, nutrition, education, and socio-economic policies, with strong political commitment and coordinated efforts across sectors. This study underscores the urgent need for integrated and context-specific strategies to combat malnutrition, improve healthcare access, and address socio-economic disparities to achieve better health outcomes for mothers and children in Madagascar.

## Figures and Tables

**Table 1:** Profile of the infant.

	Total (n = 437; 100.0%)	Healthy Weight (n = 249; 57.0%)	Stunted, Wasted, Underweight (n = 130; 29.7%)	OR	95% CI	Overweight, Obese (n = 58; 13.3%)	OR	95% CI	*p*-Value ^1^
	Frequency (%) Mean ± SD	Frequency (%) Mean ± SD	Frequency (%) Mean ± SD			Frequency (%) Mean ± SD			
**Age**	10.53 ± 6.47	10.68 ± 6.37	12.92 ± 5.58			4.53 ± 4.90			<0.001
0–6 months	137 (31.4%)	76 (30.5%)	18 (13.8%)	REFERENCE	REFERENCE	43 (74.1%)	REFERENCE	REFERENCE	0.006
7–13 months	148 (33.9%)	87 (34.9%)	51 (39.2%)	2.47	1.33–4.60	10 (17.2%)	0.20	0.10–0.43	
14–24 months	152 (34.8%)	86 (34.5%)	61 (46.9%)	2.99	1.63–5.51	5 (8.6%)	0.10	0.04–0.27	
**Sex**									0.091
Female	217 (49.7%)	119 (47.8%)	56 (43.1%)	REFERENCE	REFERENCE	31 (53.4%)	REFERENCE	REFERENCE	
Male	220 (50.3%)	130 (52.2%)	74 (56.9%)	0.69	0.45–1.06	27 (46.6%)	0.80	0.45–1.41	
**Low birth weight**									0.006
<2.5 kg	90 (20.6%)	43 (17.3%)	39 (30.0%)	2.05	1.25–3.38	8 (13.8%)	0.76	0.34-1.73	
≥2.5 kg	347 (79.4%)	206 (82.7%)	91 (70.0%)	REFERENCE	REFERENCE	50 (86.2%)	REFERENCE	REFERENCE	
**Weight** (kg)	7.31 ± 1.74	7.74 ± 1.74	6.87 ± 1.29	-	-	6.46 ± 2.07	-	-	<0.001
**Height** (cm)	66.20 ± 8.51	68.31 ± 7.93	66.96 ± 6.25	-	-	55.45 ± 7.38	-	-	<0.001
**MUAC** (mm) (N = 346)	135.91 ± 11.72	140.17 ± 9.96	127.64 ± 9.32	-	-	144.24 ± 13.94	-	-	<0.001
≤114 mm	9 (2.6%)	0 (0%)	8 (6.5%)	-	-	1 (5.9%)	-	-	<0.001
≥115 mm–≤124 mm	67 (19.4%)	14 (6.8%)	53 (43.1%)	11.72	0.09–22.57	0 (0%)	-	-	
≥125 mm normal	270 (78.0%)	192 (93.2%)	62 (50.4%)	REFERENCE	REFERENCE	16 (94.1%)	REFERENCE	REFERENCE	

CI, confidence interval; MUAC, mid-upper arm circumference; OR, odds ratio; SD, standard deviation. ^1^ *p*-value was calculated using ANOVA or Chi-squared test.

**Table 2:** Profile of the mothers.

	Total (n = 437; 100.0%)	Healthy Weight (n = 249; 57.0%)	Stunted, Wasted, Underweight (n = 130; 29.7%)	OR	95% CI	Overweight, Obese (n = 58; 13.3%)	OR	95% CI	*p*-Value ^1^
	Frequency (%) Mean ± SD	Frequency (%) Mean ± SD	Frequency (%) Mean ± SD			Frequency (%) Mean ± SD			
**Age**	10.53 ± 6.47	10.68 ± 6.37	12.92 ± 5.58	-	-	25.10 ± 5.60			0.660
<18	31 (7.1%)	15 (6.0%)	11 (8.5%)	-	-	5 (8.6%)	1.34	0.46–3.89	0.593
18–29	297 (68.0%)	173 (69.5%)	81 (62.3%)	-	-	43 (74.1%)	REFERENCE	REFERENCE	
30–39	90 (20.6%)	50 (20.1%)	31 (23.8%)	-	-	9 (15.5%)	0.72	0.33–1.59	
40–49	19 (4.3%)	11 (4.4%)	7 (5.4%)	-	-	1 (1.7%)	0.37	0.04–2.91	
**Weight (kg)**	48.61 ± 8.04	49.59 ± 8.56	46.14 ± 7.06	-	-	49.91 ± 6.60	-	-	<0.001
**Height (cm)**	152.65 ± 5.66	153.30 ± 5.96	151.14 ± 5.07	-	-	153.28 ± 5.05	-	-	0.001
**BMI (kg/m^2^)**	20.77 ± 3.05	20.95 ± 3.20	20.18 ± 2.94			21.29 ± 2.37			0.025
<18.5	108 (24.7%)	63 (25.3%)	41 (31.5%)	1.33	0.82–2.14	4 (6.9%)	0.21	0.07–0.60	0.160
18.5–24.9	289 (66.1%)	161 (64.7%)	79 (60.8%)	REFERENCE	REFERENCE	49 (84.5%)	REFERENCE	REFERENCE	
25.0–29.9	34 (7.8%)	20 (8.0%)	9(6.9%)	0.92	0.40–2.11	5 (8.6%)	0.82	0.29–2.30	
>30	6 (1.4%)	5 (2.0%)	1 (0.8%)	0.41	0.05–3.55	0 (0%)	-	-	
**Parity**									0.790
Primiparous	161 (36.8%)	94 (37.8%)	46 (35.4%)	REFERENCE	REFERENCE	21 (36.2%)	REFERENCE	REFERENCE	
2–3	203 (46.5%)	116 (46.6%)	58 (44.6%)	1.02	0.64–1.64	29 (50.0%)	1.12	0.60–2.09	
≥4	73 (16.7%)	39 (15.7%)	26 (20.0%)	1.36	0.74–2.50	8 (13.8%)	0.92	0.37–2.25	
**Twin pregnancy**									0.356
Yes	13 (3.0%)	5 (2.0%)	6 (4.6%)	2.36	0.71–3.81	2 (3.4%)	1.74	0.33–9.21	
No	424 (97.0%)	244 (99.0%)	124 (95.4%)	REFERENCE	REFERENCE	56 (96.6%)	REFERENCE	REFERENCE	
**Birth spacing**									0.018
<24 months	26 (5.9%)	14 (5.6%)	12 (9.2%)	1.71	0.77–3.81	0 (0%)	-	-	
≥24 months	411 (94.1%)	235 (94.4%)	118 (90.8%)	REFERENCE	REFERENCE	58 (100%)	REFERENCE	REFERENCE	
**Education**									0.135
Illiterate	17 (3.9%)	7 (2.8%)	7 (5.4%)	3.62	0.98–13.40	3 (5.2%)	1.38	0.29–6.48	
Primary	181 (41.4%)	94 (37.8%)	62 (47.7%)	2.39	1.02–5.57	25 (43.1%)	0.86	0.36–2.04	
Secondary 1st cycle	193 (44.2%)	119 (47.8%)	53 (40.8%)	1.61	0.69–3.76	21 (36.2%)	0.57	0.24–1.37	
Secondary 2nd cycle	46 (10.5%)	29 (11.6%)	8 (6.2%)	REFERENCE	REFERENCE	9 (15.5%)	REFERENCE	REFERENCE	
**Education level**									0.060
Primary or below	198 (45.3%)	101 (40.6%)	69 (53.1%)	1.66	1.08–2.54	28 (48.3%)	1.66	1.08–2.54	
Secondary or above	239 (54.7%)	148 (59.4%)	61 (46.9%)	REFERENCE	REFERENCE	30 (51.7%)	REFERENCE	REFERENCE	
**Occupation**									0.626
Farmer	335 (76.7%)	185 (74.3%)	108 (83.1%)	REFERENCE	REFERENCE	42 (72.4%)	REFERENCE	REFERENCE	
Seller	41 (9.4%)	27 (10.8%)	7 (5.4%)	0.44	0.19–1.05	7 (12.2%)	1.14	0.46–2.80	
Fisher	26 (5.9%)	14 (5.6%)	7 (5.4%)	0.86	0.33–2.18	5 (8.6%)	1.57	0.54–4.60	
Housewife	5 (1.1%)	2 (0.8%)	2 (1.5%)	1.71	0.24–12.33	1 (1.7%)	2.20	0.19–24.86	
Other	30 (6.9%)	21 (8.4%)	6 (4.6%)	0.49	0.19–1.25	3 (5.2%)	0.63	0.18–2.21	

BMI, body mass index; CI, confidence interval; OR, odds ratio; SD, standard deviation. ^1^ *p*-value was calculated using ANOVA or Chi-squared test.

**Table 3:** Maternal-care and feeding practices.

	Total (n = 437; 100.0%)	Healthy Weight (n = 249; 57.0%)	Stunted, Wasted, Underweight (n = 130; 29.7%)	OR	95% CI	Overweight, Obese (n = 58; 13.3%)	OR	95% CI	*p*-Value ^1^
	Frequency (%) Mean ± SD	Frequency (%) Mean ± SD	Frequency (%) Mean ± SD			Frequency (%) Mean ± SD			
**ANC**									0.410
0	3 (0.7%)	2 (0.8%)	1 (0.8%)	1.01	0.09–11.24	0 (0%)	-	-	
1	7 (1.6%)	5 (2.0%)	2 (1.5%)	0.81	0.15–4.22	0 (0%)	-	-	
2–3	31 (7.1%)	12 (4.8%)	13 (10.0%)	2.19	0.96–4.94	6 (10.3%)	2.21	0.79–6.16	
≥ 4	396 (90.6%)	230 (92.4%)	114 (87.7%)	REFERENCE	REFERENCE	52 (89.7%)	REFERENCE	REFERENCE	
**IFA supplementation**									0.376
Yes	366 (83.8%)	212 (85.1%)	104 (80.0%)	REFERENCE	REFERENCE	50 (86.2%)	REFERENCE	REFERENCE	
No	71 (16.2%)	37 (14.9%)	26 (20.0%)	1.43	0.82–2.49	8 (13.8%)	0.92	0.40–2.09	
**Type of delivery**									0.080
Vaginal	402 (92.0%)	223 (89.6%)	126 (96.9%)	REFERENCE	REFERENCE	53 (91.4%)	REFERENCE	REFERENCE	
Operative vaginal birth	23 (5.3%)	19 (7.6%)	1 (0.8%)	0.09	0.01–0.70	3 (5.2%)	0.66	0.19–2.33	
Caesarean section	12 (2.7%)	7 (2.8%)	3 (2.3%)	0.76	0.19–2.98	2 (3.4%)	1.20	0.24–5.95	
**Place of delivery**									0.382
Home	157 (35.9%)	85 (34.1%)	77 (59.2%)	1.33	0.86–2.05	19 (32.8%)	0.94	0.51–1.73	
Health center	280 (64.1%)	164 (65.9%)	77 (59.2%)	REFERENCE	REFERENCE	39 (67.2%)	REFERENCE	REFERENCE	
**Reason in case of home delivery**									0.444
Upcoming birth	72 (45.0%)	30 (55.6%)	34 (39.1%)	REFERENCE	REFERENCE	8 (42.1%)	REFERENCE	REFERENCE	
Personal choice	60 (37.5%)	13 (24.1%)	40 (46.0%)	1.00	0.34–2.94	7 (36.8%)	0.94	0.17–5.25	
Transport issues	19 (11.9%)	8 (14.8%)	9 (10.3%)	0.00	-	2 (10.5%)	-	-	
Homecare for a matron	4 (2.5%)	1 (1.9%)	2 (2.3%)	0.37	0.16–0.82	1 (5.3%)	0.74	0.24–2.26	
Lack of money	4 (2.5%)	2 (3.7%)	1 (1.1%)	0.57	0.05–6.57	1 (5.3%)	2.12	0.17–26.43	
Absence of health staff	1 (0.6%)	0 (0%)	1 (1.1%)	2.27	0.20–26.27	0 (0%)	-	-	
**Early breastfeeding initiation ^2^**									0.106
Yes	251 (57.4%)	149 (59.8%)	65 (50.0%)	REFERENCE	REFERENCE	37 (63.8%)	REFERENCE	REFERENCE	
No	186 (42.6%)	100 (40.2%)	65 (50.0%)	1.49	0.97–2.28	121 (39.4%)	0.85	0.47–1.53	
**Exclusive breastfeeding ^3^** (n = 385)									0.588
Yes	170 (44.2%)	104 (46.2%)	50 (40.3%)	REFERENCE	REFERENCE	16 (44.4%)	REFERENCE	REFERENCE	
No	215 (55.8%)	121 (53.8%)	74 (59.7%)	1.27	0.82–1.98	20 (55.6%)	1.07	0.53–2.18	
**Breastfeeding up to 1-year** (n = 304)									0.080
Yes	292 (96.1%)	166 (97.6%)	104 (92.9%)	REFERENCE	REFERENCE	22 (100%)	REFERENCE	REFERENCE	
No	12 (3.9%)	4 (2.4%)	8 (7.1%)	3.19	0.94–10.87	0 (0%)	-	-	
**Early weaning ^4^** (n = 368)									0.149
Yes	224 (60.9%)	121 (57.1%)	79 (64.2%)	1.35	0.85–2.13	24 (72.7%)	2.00	0.89–4.52	
No	144 (39.1%)	91 (42.9%)	44 (35.8%)	REFERENCE	REFERENCE	9 (27.3%)	REFERENCE	REFERENCE	
**Improved mother’s diet during pregnancy**									0.040
Yes	235 (53.8%)	142 (57.0%)	58 (44.6%)	REFERENCE	REFERENCE	35 (60.3%)	REFERENCE	REFERENCE	
No	202 (46.2%)	107 (43.0%)	72 (55.4%)	1.65	1.07–2.53	23 (39.7%)	0.87	0.49–1.56	
**Improved mother’s diet during breastfeeding**									0.379
Yes	182 (41.6%)	107 (43.0%)	48 (36.9%)	REFERENCE	REFERENCE	27 (46.6%)	REFERENCE	REFERENCE	
No	255 (58.4%)	142 (57.0%)	82 (63.1%)	1.29	0.83–1.99	31 (53.4%)	0.86	0.49–1.54	
**Iodized salt**									0.998
Yes	330 (75.5%)	188 (75.5%)	98 (75.4%)	REFERENCE	REFERENCE	44 (75.9%)	REFERENCE	REFERENCE	
No	107 (24.5%)	61 (24.5%)	32 (24.6%)	1.00	0.62–1.65	14 (24.1%)	0.98	0.50–1.91	

ANC, antenatal care; CI, confidence interval; IFA, iron and folic acid; OR, odds ratio; SD, standard deviation. ^1^ *p*-value was calculated using ANOVA or Chi-squared test. ^2^ Early breastfeeding initiation within 1 h after birth. ^3^ Exclusive breastfeeding for the first 6 months. ^4^ Early weaning before 6 months.

**Table 4:** Socio-economic profile.

	Total (n = 437; 100.0%)	Healthy Weight (n = 249; 57.0%)	Stunted, Wasted, Underweight (n = 130; 29.7%)	OR	95% CI	Overweight, Obese (n = 58; 13.3%)	OR	95% CI	*p*-Value ^1^
	Frequency (%) Mean ± SD	Frequency (%) Mean ± SD	Frequency (%) Mean ± SD			Frequency (%) Mean ± SD			
**Family size**	4.59 ± 1.69	4.5 2 ± 1.61	4.54 ± 1.53			5.05 ± 2.21			0.086
<4 persons	256 (58.6%)	151 (60.6%)	74 (56.9%)	REFERENCE	REFERENCE	31 (53.4%)	REFERENCE	REFERENCE	0.540
>4 persons	181 (41.4%)	98 (39.4%)	56 (43.1%)	1.16	0.76–1.79	27 (46.6%)	1.34	0.75–2.38	
**House dimensions**	28.81 ± 24.14	30.26 ± 27.49	27.57 ± 20.24			25.33 ± 14.65			0.290
<24	213 (48.7%)	118 (47.4%)	72 (55.4%)	1.38	0.90–2.11	34 (58.6%)	1.57	0.88–2.80	0.162
>24	224 (51.3%)	131 (52.6%)	58 (44.6%)	REFERENCE	REFERENCE	24 (41.4%)	REFERENCE	REFERENCE	
**Overcrowding ^2^ (m^2^/person)**	6.80 ± 6.54	7.35 ± 7.78	6.31 ± 4.62			5.55 ± 3.44			0.575
<5	226 (51.7%)	139 (55.8%)	70 (53.8%)	1.32	0.86–2.02	31 (53.4%)	1.45	0.82–2.57	0.908
>5	211 (48.3%)	110 (44.2%)	60 (46.2%)	REFERENCE	REFERENCE	27 (46.6%)	REFERENCE	REFERENCE	
**Middle income ^3^**									0.371
<200.000 Ar	328 (75.1%)	184 (73.9%)	103 (79.2%)	1.35	0.81–2.24	41 (70.7%)	0.85	0.45–1.60	
≥200.000 Ar	109 (24.9%)	65 (26.1%)	27 (20.8%)	REFERENCE	REFERENCE	17 (29.3%)	REFERENCE	REFERENCE	
**Source of drinking water**									0.935
Public standpipe	149 (34.1%)	84 (33.7%)	44 (33.8%)	REFERENCE	REFERENCE	21 (36.2%)	REFERENCE	REFERENCE	
Protected well	285 (65.2%)	163 (65.5%)	163 (65.5%)	0.99	0.63–1.56	37 (63.8%)	0.91	0.50–1.65	
Unprotected spring	3 (0.7%)	2 (0.8%)	2 (0.8%)	0.95	0.08–10.82	0 (0%)	-	-	
**Rice availability**									0.077
<6 months	297 (68.0%)	69 (27.7%)	48 (36.9%)	1.53	0.97–2.40	23 (39.7%)	1.71	0.95–3.11	
≥6 months	140 (32.0%)	180 (72.3%)	82 (63.1%)	REFERENCE	REFERENCE	35 (60.3%)	REFERENCE	REFERENCE	
**Toilet facility**									0.347
Yes	398 (91.1%)	231 (92.8%)	115 (88.5%)	REFERENCE	REFERENCE	52 (89.7%)	REFERENCE	REFERENCE	
No	39 (8.9%)	18 (7.2%)	15 (11.5%)	1.67	0.81–3.44	6 (10.3%)	1.48	0.56–3.91	
**Distance walking from water**	9.29 ± 11.25	8.23 ± 9.33	11.39 ± 13.66			9.09 ± 12.30			0.034
<5 min	226 (51.7%)	139 (55.8%)	60 (46.2%)	REFERENCE	REFERENCE	27 (46.6%)	REFERENCE	REFERENCE	0.141
>5 min	211 (48.3%)	110 (44.2%)	70 (53.8%)	1.47	0.96–2.26	31 (53.4%)	1.45	0.82–2.87	
**Distance walking from HC**									0.416
<45 min	233 (53.3%)	138 (55.4%)	63 (48.5%)	REFERENCE	REFERENCE	32 (55.2%)	REFERENCE	REFERENCE	
>45 min	204 (46.7%)	111 (44.6%)	67 (51.5%)	1.32	0.86–2.02	26 (44.8%)	1.01	0.57–1.79	
**Land ownership**									0.965
Yes	226 (51.7%)	130 (52.2%)	66 (50.8%)	REFERENCE	REFERENCE	30 (51.7%)	REFERENCE	REFERENCE	
No	211 (48.3%)	119 (47.8%)	64 (49.2%)	1.06	0.69–1.62	28 (48.3%)	1.02	0.58–1.80	
**Transport availability**									0.048
Yes	113 (25.9%)	73 (29.3%)	32 (24.6%)	REFERENCE	REFERENCE	8 (13.8%)	REFERENCE	REFERENCE	
No	324 (74.1%)	176 (70.7%)	98 (75.4%)	1.27	0.78–2.06	50 (86.2%)	2.59	1.17–5.74	

CI, confidence interval; HC, health center; OR, odds ratio; SD, standard deviation. ^1^ *p*-value was obtained calculated using ANOVA or Chi-squared test. ^2^ Overcrowding rate was considered as <5 m^2^ of floor area per person. ^3^ Middle income was established in national currency of Madagascar (MGA, Ariary).

**Table 5:** Minimum Dietary Diversity for Women of Reproductive Age of the mothers.

	Total (n = 437; 100.0%)	Healthy Weight (n = 249; 57.0%)	Stunted, Wasted, Underweight (n = 130; 29.7%)	OR	95% CI	Overweight, Obese (n = 58; 13.3%)	OR	95% CI	*p*-Value ^1^
	Frequency (%)	Frequency (%)	Frequency (%)			Frequency (%)			
**<5 groups**	144 (33.0%)	76 (30.5%)	50 (38.5%)	1.42	0.91–2.21	18 (31.0%)	1.02	0.55–1.90	0.280
**Grains, white roots, and tubers**	437 (100%)	249 (100%)	130 (100%)	-	-	58 (100%)	-	-	-
**Pulses**	150 (34.3%)	84 (33.7%)	50 (38.5%)	0.81	0.52–1.26	16 (27.6%)	1.33	0.71–2.52	0.330
**Nuts and seeds**	86 (19.7%)	47 (18.9%)	28 (21.5%)	0.84	0.50–1.43	11 (19.0%)	1.00	0.48–2.06	0.820
**Dairy products**	128 (29.3%)	81 (32.5%)	35 (26.9%)	1.30	0.81–2.09	12 (20.7%)	1.84	0.93–3.67	0.160
**Meat, poultry, and fish**	301 (68.9%)	179 (71.9%)	76 (58.5%)	1.81	1.16–2.83	46 (79.3%)	0.67	0.33–1.33	0.005
**Eggs**	44 (10.1%)	27 (10.8%)	10 (7.7%)	1.46	0.68–3.11	7 (12.1%)	0.88	0.36–2.14	0.540
**Dark green leafy vegetables**	322 (73.7%)	188 (75.5%)	96 (73.8%)	1.10	0.67–1.77	38 (65.5%)	1.62	0.88–2.99	0.300
**Other vitamin A-rich fruits and vegetables**	250 (57.2%)	145 (58.2%)	76 (58.5%)	1.00	0.64–1.52	29 (50.0%)	1.39	0.78–2.47	0.490
**Other vegetables**	328 (75.1%)	189 (75.9%)	94 (72.3%)	1.20	0.74–1.95	45 (77.6%)	0.91	0.46–1.80	0.660
**Other fruits**	230 (52.6%)	136 (54.6%)	66 (50.8%)	1.16	0.76–1.78	28 (48.3%)	1.29	0.73–2.28	0.600

CI, confidence interval; OR, odds ratio; SD: standard deviation. ^1^ *p*-value was obtained calculated using ANOVA or Chi-squared test.

## Data Availability

The datasets presented in this article are not readily available due to ethical personal-data-sharing restrictions. Requests to access the datasets should be directed to the corresponding author.
